# A cross-sectional study to evaluate factors related to condom use with commercial sexual partners in workers from Ecuadorian companies

**DOI:** 10.1186/s12889-015-2184-5

**Published:** 2015-09-04

**Authors:** María C. Cabezas, Marco Fornasini, Nadia Dardenne, David Barmettler, Teresa Borja, Adelin Albert

**Affiliations:** Public Health Department, University of Liège, Liège, Belgium; Medical School, Pontifical Catholic University, Quito, Ecuador; Translational Research Center, Universidad de las Américas (UDLA), Quito, Ecuador; Accreditation Canada International, Ottawa, Canada; Psychology School, Universidad San Francisco de Quito (USFQ), Cumbaya, Ecuador

## Abstract

**Background:**

Unprotected intercourse with sex workers is one of the major risk factors for HIV infection. Consistent condom use is a prerequisite to lower the incidence of HIV.

**Methods:**

We assessed the prevalence of condom use and its determinants among company workers engaged with commercial sexual partners in Ecuador. The study was based on a random sample of 115 companies and 1,732 workers stratified by province and working sector and utilized the “Behavioral Surveillance Surveys – Adult questionnaire” developed by Family Health International.

**Results:**

Of the 1,561 sexually active workers, 311 (19.9 %) reported having intercourse with sex workers. Among them 25.9 % did not use a condom at the last sexual intercourse. As for condom use frequency over the last 12 months, 29/208 (13.9 %) reported never, 23 (11.1 %) sometimes, 24 (11.5 %) almost every time and 132 (63.5 %) every time. Factors adversely affecting condom use frequency over the last 12 months were female gender (OR = 4.56, 95 % CI: 1.45-14.4), older age (OR = 1.07, 95 % CI: 1.03-1.10), low educational level (OR = 4.69, 95 % CI: 1.95-11.3) and married workers living with spouse (OR = 7.66, 95 % CI: 3.08-19.1). By contrast, factors such as age at first sexual intercourse, job category, HIV transmission and prevention measure knowledge, single workers, previous exposure to HIV intervention programs and having a casual sexual partner were not affecting condom use frequency. When considering condom use during the last sexual intercourse or during the past 12 months with commercial sexual partners, results were similar.

**Conclusions:**

Workers with low education, older age, female gender and those married living with their spouse should be targeted for specific educational interventions.

## Background

**Fig. 1:**
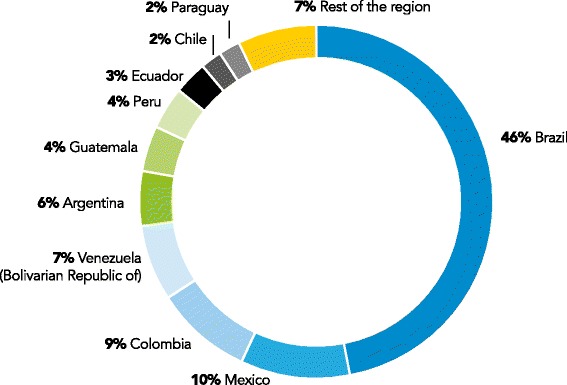
New HIV infections in Latin America, 2013. (Source: UNAIDS estimates, 2013)

Human immune deficiency virus (HIV) infection is a major health problem worldwide. According to the United Nations Programme on HIV/AIDS (UNAIDS) 2014 Fact Sheet, more than 36.9 million people live with HIV around the world and 2.1 million were newly diagnosed with HIV during 2013 [1]. In Latin America, new HIV infections have continued to grow over the years; incidence figures reported for year 2013 are displayed in Fig. 1.

Unprotected sexual intercourse with high risk sexual partners, which is still a common practice especially in countries most affected by the epidemic, represents one of the major risk factors for HIV infection [1, 2]. According to the WHO, a noteworthy decrease in HIV incidence worldwide has been associated with increased condom use [2]. UNAIDS reported that the most important factors to control the spread of the infection were sexual behavior changes; these can be most effectively prompted by improving basic knowledge about HIV/AIDS transmission pathways and prevention measures and by stressing the importance of condom use with risky sexual partners [3]. Sexual workers have been less receptive to condom use because clients generally refuse them; therefore they have come to accept unprotected sexual intercourses to avoid losing their clients [4]. Providing easy access to condoms, promoting education and HIV testing programs, however, have evidenced a positive impact by decreasing HIV prevalence in sexual workers [5, 6].

A meta-analysis related to condom use in Latinos and Latin Americans in the United States found that, along with other factors such as education level, gender, and US/Latin American nationality, condom use after an educational intervention was positively related to the knowledge regarding prevention and transmission of HIV [7]. A study in Cotonou (Benin) showed that commercial sex intercourse contributed up to 93 % (84-98 %) of all cumulative sexual infections. Further, the prevalence decline of HIV in sexual workers may have prevented 62 % (52–71 %) of new HIV infections among sexual workers between 1993 and 2008 and 33 % (20–46 %) in the overall population. These results emphasize the need of promoting safe sexual intercourses with commercial sexual partners [1].

Ecuador is a middle-income country facing health issues related to low education, poverty, gender disparities, race discrimination and religious limitations related to condom use. The Ecuadorian manual labor worker population has been among the groups most affected by HIV infection. In addition, only 1 % of HIV cases in Ecuador are not related to sexual activities [8]. A previous study of our group demonstrated a clear lack of knowledge of HIV transmission and prevention measures among a representative sample of Ecuadorian workers [9]. The present work used the same study material to estimate the prevalence of condom use and to identify factors related to condom use in Ecuadorian workers with commercial sexual partners (COSP).

## Methods

### Study design

The present study is part of a larger three-phase project (2010-2013) to improve knowledge, attitudes and sexual behavior of workers in Ecuador. It was based on a random sample of Ecuadorian companies stratified by provinces (Pichincha, Guayas and Azuay) and working sectors encompassing the greatest number of workers (commerce, manufacturing and real estate). The number of companies recorded in the National Database of Companies (*Superintendencia de Compañías de Ecuador*) was 34,917 and the corresponding number of workers was 311,050. In the database, workers are also classified into job categories (executive, administrative, manual labor and other) [10]. In each stratum, companies were selected randomly and in proportion to their actual number in the database. Workers were then selected proportionally to the actual number of workers in each working sector and category of job. This was achieved by selecting groups of approximately 14, 45 and 10 workers from each company of commerce, manufacturing and real estate, working sectors, respectively and keeping a fair representation of job categories. The actual selection of workers was as follows: for each company, the number of workers was known but no lists of names were used to guarantee anonymous selection. The company was contacted by mail or phone to request its participation in the study, and a formal invitation letter with information about the study was sent to the General Manager and Human Resources Manager. In case of refusal, another company was drawn from the database. When a company agreed to participate, workers were invited to attend a free information meeting on HIV/AIDS, where the study and its objectives were explained. After signing the informed consent form to confirm their participation in the study, participants were asked to fill in the “Family Health International” questionnaire. All questionnaires were collected by the investigators, kept anonymous and sorted by job category. In order to maintain the sample size initially planned for the company and the proportions of job categories, questionnaires in excess were discarded randomly. This procedure was applied in all 115 companies, yielding a total number of 1,732 workers. A detailed description of the selection procedure can be found in a previous publication [9]. In total, 115 companies (refusal rate: 12 %) and 1,732 workers constituted the study material (Table 1).

**Table 1:** Distribution of sampled companies and workers according to province and working sector

		Company		Worker	
		N = 115		N = 1732	
		Number	%	Number	%
Province	Pichincha	43	37.1	736	42.5
	Guayas	67	58.6	900	52.0
	Azuay	5	4.3	96	5.5
Working sector	Commerce	46	40.0	624	36.0
	Manufacturing	13	11.3	590	34.1
	Real Estate	56	48.7	518	29.9

### Subject characteristics

The socio-demographic variables collected for each participant included; gender, age (years), age at first sexual intercourse (years), education level (primary, secondary, higher), living-in partner status (married living with spouse, married living with other sexual partner, married not living with a sexual partner, single living with a sexual partner, and single not living with a sexual partner), job category and previous exposure to an educational intervention program about HIV/AIDS (yes, no) as well as HIV/AIDS transmission and prevention measures knowledge (poor, good). Data collected on the subject’s sexual activity included sexual relations over the past 12 months (yes, no) and in last sexual intercourse (vaginal or anal sex) with regular, commercial, and casual sexual partners. In each case, the estimated number of partners was noted along with condom use (yes, no) in the last sexual intercourse and frequency of condom use in the last 12 months (every time, almost every time, sometimes, never). Frequency of condom use in the last year, “consistent condom use” was defined as condom use during every sexual intercourse with a commercial sexual partner. “Inconsistent condom use” was defined as sometimes or almost every time condom use during sexual intercourse with sexual commercial partner. “Complete lack of condom use” was defined as never use condoms during every sexual intercourse with a commercial sexual partner.

### Questionnaire and study conduct

The instrument used in this study was the “Behavioral Surveillance Surveys – Adult questionnaire” developed by Family Health International in 2000. All subsequent versions of this questionnaire developed after 2000 maintained the same basic concepts and included modifications mainly based on local additions rather than significantly modifying the established questions. This questionnaire includes 4 questions about sexual history with commercial sexual partners (partners with whom they had sex in exchange for money) and casual sexual partners (non-regular, non-commercial sexual partners, not including current spouse or live-in sexual partners) [11]. Before implementing the survey, all questions were validated for language and comprehension of questions by a pilot study in Ecuador. Commercial sexual partner and casual sexual partner definitions were thoroughly explained to participants.

Questionnaires were self-administered with facilitator help and the average duration was 30 to 45 min. A surveyor manual was elaborated with the help of a sociologist to avoid bias in the survey process. The study was conducted by 10 surveyors (4 in Pichincha, 4 in Guayas and 2 in Azuay), 2 call centers and 1 study coordinator. The surveyors went through a two-day training session during which they were provided with information regarding the questionnaire and the project, and their questions were answered. The call centers staff received one-day training. Validated informed consent forms explaining the study procedures, including protocols for subject confidentiality, were signed by each participant. Electronic data records were kept anonymous and secured.

### Measurement methods

The number of commercial sexual partners and condom use in the last sexual intercourse and during the last 12 months was recorded. Sexual behavior with commercial sexual partners was assessed by questions Q501, Q503, Q506 and Q601 of the questionnaire:

- Q501: Had sexual intercourse with a commercial sexual partner (partners with whom you had sex in exchange for money) in the last 12 months.
- Q503: The last time you had sex with this commercial partner, did you and your partner use a condom?
- Q506: With what frequency did you and all of your commercial sexual partner(s) use a condom during the past 12 months?
- Q601: Had sexual intercourse with casual sexual partners in the last 12 months. “Casual sexual partners” (sexual partners that you are not married to and have never lived with and did not pay (“non-regular” partners) – Do not include current spouse(s) or live-in sexual partner).

Subjects with missing answers were not included in the statistical analysis.

### Ethics

The study was approved and supervised by The Institutional Review Board (IRB) of Universidad San Francisco de Quito in Ecuador.

### Statistical analysis

A power calculation showed that with a sample size of 1,500 workers, the prevalence of knowledge, sexual behavior, and educational needs towards HIV/AIDS could be estimated with a statistical precision of at least 3 %. Expecting a 15 % loss of data during the study conduct, a sample of 1,725 was initially required.

Quantitative variables were summarized by mean and standard deviation (SD), whereas for qualitative variables the number and percentage of subjects in each category were given. The association between each risk factor and condom use was assessed by logistic regression. Odds ratios (OR) and associated 95 % confidence intervals (95 % CI) were adjusted for the stratifying variables (province and working sector). All risks factors, potential confounders and stratifying variables were then combined into a multivariate logistic regression analysis. Likewise, ordinal logistic regression was used to assess the association of frequency of condom use (every time, almost every time, sometimes, never) in the last 12 months with the risk factors and adjusting for stratification. The strength of association between the outcome and the risk factors was assessed by the area under curve (AUC) expressed in percent; the closer AUC to 100 %, the stronger the association. Calculations were always done on the maximum number of data available, missing data were neither replaced nor imputed. Results were considered statistically significant at the 5 % critical level (P < 0.05). All calculations were done using the SAS statistical package (version 9.2 for Windows).

## Results

### Description of the study participants

Table 2 displays the characteristics of the 1,732 workers included in the study. There were 1,128 (65.1 %) males and 604 (34.9 %) females. The mean age of participants was 31.8 ± 9.0 years and 1,374 (82.9 %) participants were under 40 years of age. Most participants (n = 929, 58.6 %) were married. The education level was primary for 10.6 %, secondary for 32.7 % and higher for 56.7 % of the participants. As for the job category, a majority of the participants (50.1 %) was manual workers. Catholic religion was reported by 1,396 (80.6 %) out of 1,732 of subjects. Previous exposure to an HIV/AIDS intervention program was high: 1,103 (67.9 %) participants had been exposed and 522 (32.1 %) had not. Poor knowledge about HIV/AIDS transmission was found in 49.1 % (95 % CI: 46.6-51.6) of subjects and incorrect knowledge about preventive measures was found in 32.9 % (95 % CI: 30.6-35.2) of respondents [9].

**Table 2:** Characteristics of study participants (n = 1732 Ecuadorian workers)

Variable	Category	Number	%
Gender	Male	1128	65.1
	Female	604	34.9
Age (years)^a^	<40	1374	82.9
	>40	284	17.1
Education Level	Primary	177	10.6
	Secondary	546	32.7
	Higher	947	56.7
Marital Status	Married	929	58.6
	Single	655	41.4
Job Category	Executive	89	5.1
	Administrative	428	24.7
	Manual Labor	868	50.1
	Other	347	20.1
Previous Exposure to HIV Intervention Programs	Yes	1103	67.9
	No	522	32.1
HIV Transmission Knowledge (N = 1543)	Good	786	50.9
	Poor	757	49.1
HIV Prevention Measures Knowledge (N = 1546)	Good	1037	67.1
	Poor	509	32.9

^a^Mean age ± SD: 31.8 ± 9.0 years

### Sexual behavior

Among the 1,732 study subjects, 1,561 (90.7 %) reported to be sexually active. In sexually active subjects, only 5 (0.32 %) reported condom cost as expensive. When asked about the required time to get a condom, 816 (52.3 %) reported a mean time of less than 15 min, 64 (4.1 %) less than 1 h; 66 (4.2 %) more than 1 h and 615 (39.4 %) didn’t know the required time.

Among the 1,561 sexually active participants, 311 (19.9 %) had sexual relations with COSP. Of them, only 116 (37.3 %) accepted to be tested for HIV. The comparison of subjects with and without a commercial sexual partner is given in Table 3. Having a commercial sexual partner was significantly associated with; male gender, young age at first sexual intercourse, manual labor job category, low education level, poor HIV transmission and prevention measures knowledge, lack of previous exposure to HIV prevention programs and having a casual sexual partner. No significant difference was found for age and living-in status.

**Table 3:** Comparison of sexually active subjects with and without commercial sexual partners (COSP)

			COSP		
Variable	Category	N	Yes	No	P-value*
Number (%)		1561	311 (19.9)	1250 (80.1)	
Gender		1561			<.0001
	Male		284 (26.5)	788 (73.5)	
	Female		27 (5.5)	462 (94.5)	
Age (years)	Mean ± SD	1561	32.1 ± 9.7	31.8 ± 8.5	0.67
Age at first sexual intercourse (years)	Mean ± SD	1561	15.6 ± 2.5	17.4 ± 3.6	<.0001
Level of education		1504			<.0001
	Primary		57 (37.3)	96 (62.7)	
	Secondary		124 (24.7)	378 (75.3)	
	Higher		112 (13.2)	737 (86.8)	
Living in status		1434			0.16
	A*		138 (17.6)	647 (82.4)	
	B*		13 (22.8)	44 (77.2)	
	C*		17 (26.6)	47 (73.4)	
	D*		36 (17.5)	170 (82.5)	
	E*		70 (21.7)	252 (78.3)	
Job category		1561			<.0001
	Executive		13 (15.7)	70 (84.3)	
	Administrative		45 (11.7)	340 (88.3)	
	Manual Labor		199 (25.5)	583 (74.5)	
	Other		54 (17.4)	257 (82.6)	
Previous exposure to HIV Prevention Programs		1469			0.0014
	Yes		162 (16.2)	836 (83.8)	
	No		116 (24.6)	355 (75.4)	
HIV Transmission knowledge		1392			<.0001
	Good		96 (13.5)	616 (86.5)	
	Poor		154 (22.7)	526 (77.3)	
HIV Prevention measure knowledge		1394			0.031
	Good		149 (15.7)	800 (84.3)	
	Poor		93 (20.9)	352 (79.1)	
Casual Sexual Partner		1561			<.0001
	Yes		199 (40.7)	290 (59.3)	
	No		112 (10.5)	960 (89.5)	

A* Married, living with spouse

B* Married, living with other sexual partner

C* Married, not living with a sexual partner

D* Not married, living with a sexual partner

E* Not married, not living with a sexual partner

*Univariate logistic regression but adjusted for province and working sector

### Condom use

Among the 239 subjects with a commercial sexual partner who provided information about condom use during the last sexual intercourse, 177 (74.1 %) indicated they used a condom and 62 (25.9 %) did not. As for the 208 subjects with a commercial sexual partner who reported about the frequency of condom use during the last 12 months, the distribution was as follows: every time (63.5 %), almost every time (11.5 %), sometimes (11.1 %) and never (13.9 %). Thus, consistent condom use amounted 63.5 % (95 % CI: 57.0-70.0), inconsistent condom use 22.6 % (95 % CI: 16.9-28.3) and complete lack of condom use 13.9 (95 % CI: 9.19-18.6). When cross-classifying condom use and frequency of condom use (see Table 4), a highly significant association was found between the two responses (P < 0.0001).

**Table 4:** Relationship between and frequency of condom use during last 12 months and condom use of last sexual intercourse with commercial sexual partners

Last sexual intercourse condom use	Frequency of condom use last 12 months				
	Every time	Almost every time	Sometimes	Never	Total
	N (%)	N (%)	N (%)	N (%)	N (%)
Yes	122 (95.3)	18 (94.7)	9 (36.0)	1 (4.0)	150 (76.9)
No	6 (4.7)	1 (5.3)	14 (64.0)	24 (96.0)	45 (23.1)
Total	128 (100)	19 (100)	23 (100)	25 (100)	195 (100)

P < 0.0001

Unprotected last sexual intercourse with a commercial sexual partner was significantly associated with; female gender (OR = 7.16, 95 % CI: 2.34-21.9), older age (OR = 1.06, 95 % CI: 1.02-1.10), low level of education (OR = 3.91, 95 % CI: 1.53-9.98), and married workers living spouse (OR = 6.82, 95 % CI: 2.48-18.7) or living with other sexual partner (OR = 16.5, 95 % CI: 3.71-73.4) as compared to single living subjects. Factors, such as age at the first sexual intercourse, job category, previous exposure to HIV prevention programs, HIV transmission and prevention measures knowledge, and having a casual sexual partner were not related to condom use during the last sexual intercourse with commercial sexual partners (see Table 5). When combining all variables into a multivariate logistic regression analysis and adjusting for stratification factors, only female gender, young age, living-in status with spouse or other sexual partner and to a lesser extent low education remained significant. The strength of association between condom use and risk factors amounted 84.7 %.

**Table 5:** Factors related to condom use during the last sexual intercourse with a commercial sexual partner

			Condom use at last sexual intercourse				
Variable	Category	N	No	Yes	OR (95 % CI)*	P-value*	P-value**
Number (%)		239	62 (25.9)	177 (74.1)			
Gender		239				0.0006	0.0005
	Male		51 (22.9)	172 (77.1)	1.0		
	Female		11 (68.7)	5 (31.3)	7.16 (2.34 – 21.9)		
Age (years)	Mean ± SD	223	34.8 ± 10.1	30.1 ± 8.1	1.06 (1.02 – 1.10)	0.0014	0.079
Age of first sexual intercourse (years)	Mean ± SD	224	15.8 ± 2.7	15.5 ± 2.4	1.05 (0.93 – 1.18)	0.45	0.63
Level of education		229				0.016	0.051
	Primary		16 (38.1)	26 (61.9)	3.91 (1.53 – 9.98)		
	Secondary		25 (24.5)	77 (75.5)	1.71 (0.81 – 3.63)		
	Higher		15 (17.6)	70 (82.4)	1.0		
Living in status		212				0.0005	0.042
	A*		37 (36.6)	64 (63.4)	6.82 (2.48 – 18.7)		
	B*		7 (58.3)	5 (41.7)	16.5 (3.71 – 73.4)		
	C*		4 (30.8)	9 (69.2)	4.34 (0.96 – 19.6)		
	D*		4 (16.0)	21 (84.0)	2.21 (0.54 – 9.19)		
	E*		5 (8.2)	56 (91.8)	1.0		
Job category		239				0.10	0.96
	Executive		3 (37.5)	5 (62.5)	1.0		
	Administrative		5 (14.3)	30 (85.7)	0.25 (0.04 – 1.45)		
	Manual Labor		47 (30.3)	108 (69.7)	0.67 (0.15 – 3.00)		
	Other		7 (17.1)	34 (82.9)	0.31 (0.06 – 1.67)		
Previous exposure to HIV prevention programs		227				0.57	0.83
	Yes		32 (24.1)	101 (75.9)	1.0		
	No		26 (27.7)	68 (72.3)	0.84 (0.45 – 1.57)		
HIV transmission knowledge		200				0.86	0.52
	Good		18 (22.8)	61 (77.2)	1.0		
	Poor		30 (24.8)	91 (75.2)	1.06 (0.51 – 2.19)		
HIV prevention measures knowledge		201				0.18	0.82
	Good		29 (22.7)	99 (77.3)	1.0		
	Poor		23 (31.5)	50 (68.5)	1.55 (0.81 – 2.97)		
Casual sexual partner		239				0.93	0.75
	Yes		38 (25.8)	109 (74.2)	1.03 (0.56 – 1.88)		
	No		24 (26.1)	68 (73.9)	1.0		

A* Married, living with spouse

B* Married, living with other sexual partner

C* Married, not living with a sexual partner

D* Not married, living with a sexual partner

E* Not married, not living with a sexual partner

*Univariate logistic regression but adjusted for province and working sector

**Multivariate logistic regression adjusted for province and working sector (N = 187) – AUC = 84.7 %

### Frequency of condom use

When assessing the association between frequency of condom use with commercial sexual partners in the last 12 months with the various risk factors, results were similar to those obtained for condom use during the last sexual intercourse. Specifically, females were at higher risk than males (OR = 4.56, 95 % CI: 1.45-14.4). The risk of inconsistent condom use increased with age (OR = 1.07, 95 % CI: 1.03-1.10), low educational level (OR = 4.69, 95 % CI: 1.95-11.3) and with living-in status in the sense that, in all subject categories, the odds ratios (range: 4.48 to 7.66) were markedly higher than in the single living-in individuals. Factors such as age at first sexual intercourse, job category, HIV transmission and prevention knowledge, previous exposure to HIV prevention programs and having a casual sexual partner were not related to condom use frequency during the last 12 months with commercial sexual partners (see Table 6). When combining all variables into a multivariate ordinal logistic regression analysis and adjusting for stratification factors, only female gender, low level of education, non-single living-in status and to a lesser extent age were significant. The strength of association between frequency of condom use and risk factors assessed by the AUC was equal to 66.9 %.

**Table 6:** Factors related to frequency of condom use with commercial sexual partners in the last 12 months

			Frequency of condom use						
Variable	Category	N	Every time	Almost every time	Sometimes	Never	OR (95 % CI)*	P-value*	P-value**
Number (%)		208	132 (63.5)	24 (11.5)	23 (11.1)	29 (13.9)			
Gender		208						0.0095	0.0041
	Male		129 (65.5)	24 (12.2)	19 (9.6)	25 (12.7)	1.0		
	Female		3 (27.3)	0 (0.0)	4 (36.4)	4 (36.4)	4.56 (1.45 – 14.4)		
Age (years)	Mean ± SD	208	29.0 ± 7.8	30.5 ± 8.3	36.1 ± 12.2	36.3 ± 10.5	1.07 (1.03 – 1.10)	0.0001	0.053
Age at first sexual intercourse (years)	Mean ± SD	208	15.3 ± 2.4	15.3 ± 1.7	15.9 ± 2.8	15.7 ± 2.6	1.09 (0.96 – 1.22)	0.17	0.15
Level of education		199						0.0009	0.0055
	Primary		16 (41.0)	5 (12.8)	7 (18.0)	11 (28.2)	4.69 (1.95 – 11.3)		
	Secondary		58 (67.4)	12 (14.0)	10 (11.6)	6 (7.0)	1.31 (0.63 – 2.72)		
	Higher		56 (75.7)	4 (5.4)	5 (6.8)	9 (12.1)	1.0		
Living in Status		182						0.0006	0.021
	A*		42 (50.0)	11 (13.1)	14 (16.7)	17 (20.2)	7.66 (3.08 – 19.1)		
	B*		3 (50.0)	2 (33.3)	0 (0.0)	1 (16.7)	4.48 (0.77 – 26.0)		
	C*		9 (56.3)	1 (6.2)	0 (0.0)	6 (37.5)	7.13 (2.07 – 24.6)		
	D*		12 (57.1)	3 (14.3)	2 (9.5)	4 (19.1)	5.81 (1.79 – 18.9)		
	E*		48 (87.2)	3 (5.5)	3 (5.5)	1 (1.8)	1.0		
Job category		208						0.44	0.34
	Executive		5 (71.4)	0 (0.0)	0 (0.0)	2 (28.6)	1.0		
	Administrative		22 (75.8)	1 (3.5)	1 (3.5)	5 (17.2)	0.64 (0.11 – 3.70)		
	Manual Labor		80 (59.7)	17 (12.7)	17 (12.7)	20 (14.9)	1.23 (0.25 – 6.06)		
	Other		25 (65.8)	6 (15.8)	5 (13.1)	2 (5.3)	0.85 (0.15 – 4.62)		
Previous exposure to HIV Prevention Programs		197						0.97	0.86
	Yes		72 (63.2)	18 (15.8)	13 (11.4)	11 (9.7)	1.0		
	No		55 (66.3)	6 (7.2)	7 (8.4)	15 (18.1)	0.99 (0.54 – 1.82)		
HIV Transmission knowledge		180						0.19	0.47
	Good		46 (64.8)	6 (8.5)	8 (11.2)	11 (15.5)	1.0		
	Poor		74 (67.9)	16 (14.7)	7 (6.4)	12 (11.0)	0.63 (0.32 – 1.27)		
HIV Prevention knowledge		175						0.50	0.34
	Good		74 (67.3)	14 (12.7)	12 (10.9)	10 (9.1)	1.0		
	Poor		42 (64.6)	5 (7.7)	5 (7.7)	13 (20.0)	1.23 (0.66 – 2.33)		
Casual Sexual Partner		208						0.15	0.78
	Yes		80 (59.3)	20 (14.8)	16 (11.8)	19 (14.1)	1.56 (0.84 – 2.88)		
	No		52 (71.2)	4 (5.5)	7 (9.6)	10 (13.7)	1.0		

A* Married, living with spouse

B* Married, living with other sexual partner

C* Married, not living with a sexual partner

D* Not married, living with a sexual partner

E* Not married, not living with a sexual partner

*Univariate logistic regression but adjusted for province and working sector

**Multivariate logistic regression adjusted for province and working sector (N = 165) – AUC = 66.9 %

## Discussion

The present study focused on sexually active workers who declared having sexual intercourse with COSP. In terms of HIV/AIDS, COSP is one of the highest risk groups of propagating the epidemic. Subjects with a COSP reported low socio-economic conditions, poor HIV transmission and prevention measures knowledge and an early age of sexual initiation. We found that 25.6 % of subjects did not use condom in their last sexual intercourse with a COSP. When looking at the last 12 months, complete lack of condom use was 13.9 % while inconsistent condom use amounted 22.6 %.

Interestingly, the present study showed that the risk factors characterizing subjects having sexual intercourse with a COSP were not necessarily the same as those related to condom use in such individuals. Indeed, characteristics such as male gender, younger age at first sexual intercourse, manual labor, non-exposure to previous HIV prevention programs, lack of HIV transmission and prevention knowledge, and having a casual sexual partner described the typical pattern of “COSP subjects”. By contrast, factors like female gender (despite the small number of women in the high-risk group), older age, and married but living with another sexual partner, were affecting condom use in a negative way. The only factor common to the two outcomes was low educational level.

The figures obtained respectively for lack of condom use in the last sexual intercourse and for inconsistent condom use in the last 12 months with commercial sexual partners were comparatively high to those found in other studies. In an Indian study, only 19 % of subjects used a condom during intercourse with their non-regular sexual partners compared with 63.5 % in our study [12]. Another study in Bolivia on truck drivers reported that 31 % of them never used condoms while 43 % used them within the last month. The latter percentages are somewhat worse than ours but truck drivers are known to be particularly at risk by travelling across large areas all year long [13].

In Ecuador, only few studies have focused on the number and type of sexual partners of the sexually active population. A survey of university students in 2007 reported that, among the 219 sexually active, 44.8 % of males and 5.4 % females had more than one sexual partner at that time. The study however did not specify the type of sexual partners. Compared with the present study, males from that study had more sexual partners [14].

A study of prisoners of both genders in Ecuador in 2008 reported that 35.5 % of subjects had more than one sexual partner in the last 12 months, a percentage similar to that of the present study (34.5 %) [14]. The study of commercial sexual partners in Ecuador in 2009 reported that 97.2 % of them used a condom with their last client. By contrast, in the present study, 74.1 % of participants reported condom use with their last COSP. Another study in men who have sex with men in Ecuador showed that 55 % had unprotected penetrative sex with their last three sexual partners [15].

In another study among brewery workers in Nigeria with similar socio-demographical profile as those from the present study reported a consistent condom use in 12 %, which contrasts with the present study that found condom use to be higher [16]. A study on young male workers in Mexico has shown that psychosocial and psychosexual factors may influence the self-efficacy for condom use among male clients with sexual workers [17]. Thus, to efficiently promote consistent condom use, the present findings underline the need to understand the workers vulnerability, their sexual practices, the experienced barriers to condom use, and their knowledge and related attitudes towards HIV [18, 19].

This study has a number of limitations. The database of the “Superintendencia de Compañías de Ecuador” does not contain all existing companies in the country; therefore, informal companies have not been accounted for. It is clear that the material and financial resources of the companies and their working sector (commerce, manufacturing or real estate) influence the educational level of their employees. Therefore, the proportion of workers with a low education level in the sample is most likely underestimated. The study has been limited to three provinces; Pichincha, Guayas and Azuay. Although they are the most important provinces, the other provinces may exhibit differences regarding risk factors associated with lack of condom use. Another limitation is the lack of information regarding the gender of COSP and their sexual behavior. The study defined commercial sexual partners with whom they had sex in exchange for money not for other goods. Therefore, it is likely that there were male and female sexual workers. In addition, important variables such urban and rural location, ethnicity and nationality and transgender of participants were not registered.

To the best of our knowledge, the present study is the first to look at factors associated with condom use (or lack thereof) in occupational settings in Ecuador. It is worth mentioning that in 2003 UNAIDS already prepared a document for the business and industrial sectors emphasizing the importance of implementing prevention programs in occupational settings [20]. The WHO recommended that companies implement HIV/AIDS policies and prevention programs because it is a favorable investment to avoid discrimination and prevent HIV/AIDS in occupational settings.

In Latin America there are only a few studies targeting the working populations. Studies in healthcare workers with a focus on HIV/AIDS prevention and transmission knowledge have shown a poor knowledge of HIV transmission/prevention. However, they did not provide information on factors related to condom use [21–23]. Thus, the results of this study should trigger other initiatives in the domain.

National reports have persistently indicated that manual labor workers have been one of the most affected groups by the HIV epidemic in Ecuador. In this context, wider delivery of effective behavior change strategies to promote protected sexual intercourse with risky sexual partners is central to reversing the global HIV epidemic. Human behavior will remain critical as new prevention strategies are unlikely to be 100 % effective in preventing transmission [24]. Therefore, the HIV/AIDS epidemic remains a serious health challenge worldwide; in countries where the epidemic continues to expand as it is the case for Ecuador, there is an urgent need to pursue the current effective strategies to promote safer behaviors [1]. In this perspective, an educational video specifically dedicated to companies was developed to promote HIV-related knowledge, attitudes and prevention for company workers [25]. Unfortunately, as alluded to in a recent study [17], correlates of efficacy for condom use remain largely unstudied because behavioural change theories presume that self-efficacy predicts condom use.

## Conclusions

The present study demonstrates a major lack of condom use among workers having sexual intercourse with commercial sexual partners in Ecuador. Having a commercial sexual partner is influenced by factors such as young age at the first sexual intercourse, male gender, low education level, poor HIV transmission and prevention knowledge, lack of previous exposure to HIV prevention programs and having a casual sexual partner. Condom use with COSP was negatively influenced by factors such as old age, female gender, low education level and married status of living with a spouse or other sexual partner. Workers with low education level, older age, female gender and married should be targeted for specific interventions emphasizing the importance of consistent condom use with commercial sexual partners. The study stresses the need for more evaluations and educational intervention programs to reduce HIV/AIDS transmission in companies in Ecuador.
